# Early Onset Priapism Under Chlorpromazine and Risperidone Therapy

**Published:** 2011

**Authors:** Mahin Eslami Shahrbabaki, Laya Sabzevari

**Affiliations:** 1 Department of psychiatry, Kerman university of medical sciences, Shahid Beheshti psychiatric hospital

**Keywords:** Chlorpromazine, Priapism, Risperidone

## Abstract

Priapism is a prolonged and usually painful erection which is not associated with the sexual desire. It is an uncommon urologic emergency with variety of known etiologies such as the use of psychotropic medications.

Priapism under concurrent treatment with chlorpromazine and risperidone has not been reported so far. Herein, a psychotic patient who developed priapism during chlorpromazine and risperidone therapy will be reported.

## Introduction

Priapism is considered as a true urologic emergency .Two types of priapism have been described: veno- occlusive (low-flow) or ischemic and arterial (high-flow) or non-ischemic. High-flow priapism occurs secondary to penile or perineal trauma while low–flow priapism may result from sickle cell disease, leukemia, anticoagulants, spinal cord lesions and drugs (1,2).

Antipsychotic agents were implicated in 15% to 26% of priapism associated with medications (3). The mechanism of drug–induced priapism is unknown. However, it is now recognized that psychotropic agents may have peripheral alpha-1 blocking activity and/or central serotonin–like activity (1). Drugs including thiothexene, chlorpromazine, thioridazine and risperidone have a relatively high alpha-1 adrenergic activity as accusative factor of priapism (4). Trazodone and phenoxybenzamine have also alpha-1 blocking properties and may cause priapism (5). Chlorpromazine is a conventional antipsychotic which is more used for its sedative properties. Risperidone is an atypical antipsychotic agent that is preferred in medical treatment of psychotic patients because of its lower extra pyramidal side effects.

Both of these antipsychotic agents have alpha-1 antagonist activity. Until now, dose and duration specific antipsychotic-induced priapism has not been reported. However, priapism is a rare sequel of antipsychotics. It is considered as an urologic urgency and 40-50 % of these patients become impotent even after surgical treatments. Therefore, clinicians should be familiar with this infrequent and serious antipsychotic side effect and inform patients about priapism signs. A patient with priapism attributed to chlorpromazine and risperidone will be described here.

## Case Report

A 35-year–old man, divorced, educated till the 8 the grade, jobless, from Kerman was admitted to Kerman shahid Beheshti psychiatric hospital for the first time. His illness had started 20years prior to his admission but his symptoms had become worse and significant seven months before admission. He had persecutory delusions and delusions of being controlled. He had auditory hallucinations, self talking and insomnia, but no visual or other hallucinations. Besides, he was restless at nights. He had been hyper-religious and aggressive and believed that his hospital admission had been recommended by God. In his past medical history, a history of tonic-colonicseizure after tramadol abuse 3 years before admission was reported by his family. In personal history, he had left school at 8th grade. Furthermore, he had risky driving and aggressive behaviors and changed his vocations frequently. He got married 15 years and divorced 11 years prior to his admission. He had been socially isolated and had self-mutilating, suicidal attempts and tattoo history. What was observed on admission day? A wound on his scalp with dressing after struggle with his family members. He was agitated and had defensive attitude, euthymic mood, inappropriate and depressed affect. He was alert and had an intact orientation to time/ person/ place, impaired attention, normal fund of knowledge. His insight was poor. With the diagnosis of schizophrenia and a borderline personally disorder 3mg/day risperidone, clonazepam 1mg at night, biperiden 4mg/dayin two divided doses were prescribed. On the 7th day of admission due to continuing his complaint about insomnia, chlorpromazine 50mg at night was administrated. He developed priapism after 3 days latency in his painful erection announcement, seven and 13 days after prescription of chlorpromazine and risperidone respectively. After an urgent urologist consultation, aspiration of blood from corpus cavernosum was done which was failed so proximal penile shunt was done for him. Unfortunately, he had semi-rigid penis postoperatively and became candidate for penectomy.

## Discussion

Although priapism is a rare disease, all physicians should be aware of the necessity of early intervention and appropriate management in such cases. The prevalence of drug–induced priapism has increased because of an increased rate of drug use and abuse (6). The mechanism of drug-induced priapism is unknown. Antihypertensive drugs such as hydralazine, guanethidine and prazosin, antipsychotic drugs of the phenothiazine group, especially chlorpromazine and antidepressants have been associated with prolonged erection. Although millions of men take antipsychotics, antidepressants orantihypertensive agents, only a few develop priapism. The dose of clinical use does not seem to influence the probability of priapism significantly. Although the dose of chlorpromazine that causes priapism is controversial, it is not considered to be a dose–specific complication (5). Dawson–Butterworth described an idiopathic priapism associated with schizophrenia in 1969, obtaining relief from an agent within the same group of drugs as chlorpromazine. There were no previous reports of priapism on phenothiazine therapy in 1969. Therefore, it was called idiopathic priapism (7). Today, we know that psychotropic medications are the most common causes of drug–associated priapism (8,9,10). In Merkin’s report (1977), the patient who had been treated with single chlorpromazine intramuscular injection for remitting his hiccups developed priapism. Kliciler et al (2003) presented a 30-year-old man with priapism for 8 hours. He had been receiving just chlorpromazine for chronic schizophrenia for 3 years. This shows that priapism can occur after a long term therapy with an antipsychotic like chlorpromazine and supports the fact that priapism is not a dose-specific complication (11).

In order to investigate the mechanism of drug-induced priapism, Abber et al (1987) injected trazodone and chlorpromazine intracorporeally and intravenously into fourteen dogs. They observed that *intravenous*trazodone and chlorpromazine could not lead to the penile erection. However, their interracial injection resulted inpenile erection (12). Besides, they believe that local *trazodone* and chlorpromazine alpha-1antagonist properties probably result in priapism.

From the conventional antipsychotic agents, chlorpromazine and thioridazine have the greatest alpha adrenergic affinity and have been most frequently reported to be associated with priapism (13,14). Of atypical antipsychotics, risperidone has been reported to have a greater alpha adrenergic affinity, although 3 of 5 currently U.S. Food and Drug Administration (FDA)–approved atypical antipsychotics have been reported to be associated with priapism (15).

Numerous priapism cases have been reported in patient sreceiving conventional antipsychotics, especially the phenothiazines (16). More recently, three of the five atypicalanti psychotics approved by FDA for treatment of psychosis have been reported to be associated with priapism invarious case reports (17). For example, priapism associated with clozapine was first reported in 1992 (18,19,20). Since then, several other cases have been reported such as three patients with risperidone–associated priapism (15) or four patients treated with olanzapin. However, *quetiapine *has not been reported to cause priapism (14). A 22-year-old African-American man developed priapism during treatment with risperidone and on a later occasion during treatment with ziprasidone (14).Virtually, all antipsychotic medications have been reported to rarely cause urologic emergency. Clinicians should be familiar with infrequent serious adverse events of antipsychotic medications. In several reports, patients had experienced prolongederections once started on chlorpromazine therapy (15-16). In one case report, priapism occurred 5 days after a single intramuscular injection of chlorpromazine (17). In another case report, priapism occurred after the patient had taken only one 25-mg tablet of chlorpromazine, believed to be the lowest dose to produce this complication (11). Priapism occurring in a patient after inserting a crushed 25 mgchlorpromazine tablet into the urethral meatus of his penis, an unusual cause of priapism, has also been reported (21). So far, chlorpromazine has only shown an effect after local application (22). It is found that the *intracorporeal* injection of chlorpromazine produces an erection similar topapaverine in dogs, showing the anti-alpha-adrenergic properties of this drug. A study mentioned that Chlorpromazine induced priapism can occur within 28 days of initiation of drug therapy (23). In our case, the patient had been receiving 3 mg risperidone and 50 mg chlorpromazine 13 and 7 days before the priapism respectively. This shows that priapism can occur after a short-term antipsychotic therapy and supports that priapism is not a dose-specific side effect. On the other hand, concurrent use of these two drugs may be associated with an increased risk of priapism.

As a result, based on this data, priapism is not a dose-or duration-specific complication. The physician prescribing medications associated with priapism should be aware of a history of prolonged erections and patients should be informed about this complication. Patients, especially those using antipsychotics, may not mention their painful penile erection as they have psychiatric disorders. Hence, certain patients may be more vulnerable than others to this adverse effect. Even after the surgical treatment of priapism, 40-50% of patients who develop priapism become impotent (15). Therefore, the physician should be very careful about a possible history of prolonged erection and should perform a complete physical examination in order to give an appropriate medical help in cases of priapism. Moreover, we suggest that concurrent use of antipsychotics should be prevented, especially those with high alpha-one blocking activities.

## Authors' contributions

The first author have been involved in the acquisition of clinical data and in the reviewing the scientific literature and writing the manuscript. The second author followed up the case, performed complementary interview with patient, participated in literature review, evaluated the paraclinical assessments, contributed to the final version and carried out the clinical case report. Both authors read and approved the final manuscript.

## Notes

**Declaration of interest**: None.
